# Dynamic arrest and aging of biomolecular condensates are modulated by low-complexity domains, RNA and biochemical activity

**DOI:** 10.1038/s41467-022-30521-2

**Published:** 2022-05-31

**Authors:** Miriam Linsenmeier, Maria Hondele, Fulvio Grigolato, Eleonora Secchi, Karsten Weis, Paolo Arosio

**Affiliations:** 1 Department of Chemistry and Applied Biosciences, Institute for Chemical and Bioengineering, Swiss Federal Institute of Technology, 8093 Zurich, Switzerland; 2 Department of Biology, Institute for Biochemistry, Swiss Federal Institute of Technology, 8093 Zurich, Switzerland; 3 Biozentrum, Center for Molecular Life Sciences, University of Basel, 4056 Basel, Switzerland; 4 Department of Civil, Environmental and Geomatic Engineering, Swiss Federal Institute of Technology, 8093 Zurich, Switzerland

**Keywords:** Supramolecular assembly, Biophysical chemistry, RNA metabolism

## Abstract

Biomolecular condensates require suitable control of material properties for their function. Here we apply Differential Dynamic Microscopy (DDM) to probe the material properties of an in vitro model of processing bodies consisting of out-of-equilibrium condensates formed by the DEAD-box ATPase Dhh1 in the presence of ATP and RNA. By applying this single-droplet technique we show that condensates within the same population exhibit a distribution of material properties, which are regulated on several levels. Removal of the low-complexity domains (LCDs) of the protein decreases the fluidity of the condensates. Structured RNA leads to a larger fraction of dynamically arrested condensates with respect to unstructured polyuridylic acid (polyU). Promotion of the enzymatic ATPase activity of Dhh1 reduces aging of the condensates and the formation of arrested structures, indicating that biochemical activity and material turnover can maintain fluid-like properties over time.

## Abstract (web summary)

Here the authors analyze material properties and aging of active phase-separated condensates by Differential Dynamic Microscopy. Arrested states are promoted by structured RNA. Low-complexity domains and biochemical reaction keep the droplets fluid-like.

## Introduction

The ability of cells to form compartments is crucial to coordinate a variety of reactions in space and time. In addition to membrane-bound compartments, it is becoming increasingly clear that cells form membraneless organelles by liquid–liquid phase separation (LLPS) of proteins and nucleic acids^1–4^. The dynamic formation and dissolution of these biomolecular condensates is governed by a variety of intermolecular interactions^5^, which involve multivalency and repetitive sequence patterns^6–9^. These multivalent interactions can be promoted by intrinsically disordered protein sequences known as low-complexity domains (LCDs)^10^, by globular protein–protein interactions^8^ or by RNA–protein interactions^11^. An important feature of phase separating systems is the responsiveness to changes in ionic strength and pH, but also factors like ATP^12,13^, nucleic acids^14–19^, and small molecules^20^.

While a lot of attention has been dedicated to the effect of different factors on the formation and dissolution of biomolecular condensates, mechanisms that control their material properties have remained much less explored. Yet, suitable material properties (viscosity, elasticity, surface tension) are likely crucial for the proper physiological function of biomolecular condensates and misregulation of these properties may lead to pathologies^21^.

Biological condensates contain molecular networks whose formation is mediated by multivalent interactions^6,22^ and can therefore be considered as structured network fluids^23^. A variety of material properties ranging from liquid-like to dynamically arrested gel- or glass-like have been reported. In some cases, maturation from a liquid-like state into such arrested states has been observed over time^24,25^, potentially leading to the formation of aberrant protein aggregates or amyloids. This pathological liquid-to-solid phase transition has been associated with neurodegenerative diseases^26–30^. Understanding the regulation of the material properties after condensate formation and their evolution over time is particularly important. For instance, for condensates hosting biochemical reactions, fluidity is typically required to recruit client molecules and rapidly release products after processing^31^. By contrast, other condensates may require a certain level of rigidity to form a stable structural matrix^24,32–35^. The assessment of the material properties of condensates, especially of dynamically arrested states, is still very limited in vivo and only a few methods are recently emerging in vitro^36–38^. In addition, the molecular factors that modulate these material properties have remained largely unraveled.

Techniques capable to probe the dynamics of the systems are ideal to distinguish between liquid-like and gel-/glass-like materials^39^. In this context, several techniques have been developed in soft matter physics, including particle tracking and optical tweezers^17,36,37,40,41^.

Here, we apply differential dynamic microscopy (DDM) to probe the material properties of in vitro models of biomolecular condensates. DDM probes the microscopic dynamics of the condensates by monitoring fluctuations in the intensity of scattered light over time. The technique is not invasive and can be applied in combination with nanoparticle tracers with size below the optical resolution. This allows us to probe also small condensates that would exclude the large particle tracers that are conventionally required for particle tracking experiments^42^. A key advantage of the technique consists in the possibility to analyze individual condensates with sizes ranging from a few to hundreds of microns, therefore providing information on the distribution of material properties within a population of condensates. Moreover, DDM can be performed on a simple widefield microscope in brightfield mode without the requirement of additional equipment. The technique provides an attractive opportunity to probe the material properties of condensates as a function of several molecular determinants over time.

In this work, we apply DDM to investigate the material properties of condensates formed by the P-body-associated DEAD-box ATPase Dhh1 depending on LLPS-relevant factors such as ATP and RNA. This protein has several interesting features to explore the relationship between biochemical activity, dynamics of formation and dissolution of the condensates and their material properties.

**Fig. 1:**
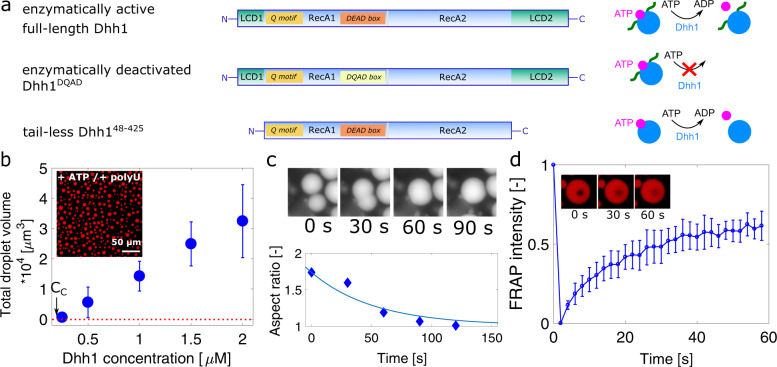
In vitro phase separation of Dhh1. **a** Dhh1 variants used in this study: full-length Dhh1, consisting of two RecA domains as core, flanked by an N- and a C-terminal low-complexity domain (LCD1/2). Q motif and DEAD box are important motifs to bind and hydrolyze ATP. In the Dhh1^DQAD^ variant, a single glutamate has been substituted with a glutamine, leading to an ATP hydrolysis-deficient variant, while the tail-less Dhh1^48–425^ construct lacks both the N- and C-terminal LCD. **b** Effect of Dhh1 concentration on droplet formation. Dhh1 solutions at increasing protein concentrations were supplied with 5 mM ATP/MgCl_2_ and 0.5 mg/ml polyU and imaged using widefield fluorescence microscopy providing a critical concentration (C_C_) equal to 0.2 µM. The panel shows the average total droplet volume of three different samples, with error bars representing the standard deviation. **c** Fusion and relaxation of two representative droplets into one single droplet, quantified by measuring the aspect ratio of two fusing droplets over time. **d** Mean FRAP signal of three different droplets of the droplets shown in (**b**), exhibiting recovery of 61 ± 2%. Error bars represent the standard deviation of the FRAP signal of three different droplets. Source data for panels **b**–**d** are provided in the Source Data file.

Dhh1 has a globular core consisting of two RecA-like domains that contain the binding sites for RNA and ATP. These core domains are connected by a linker and are flanked by two LCDs^12,43,44^ (Fig. 1a).

While ATP binding and hydrolysis are mediated by the DEAD-box sequence and the Q motif^45^, the interaction with RNA is established via electrostatic interactions between the phosphate backbone of the RNA molecule and a positively charged cleft on the two RecA domains^43^. The binding of Dhh1 to ATP and RNA has been shown to be important to promote the formation of both P-bodies in vivo and reconstituted liquid-like droplets in vitro. Furthermore, ATP hydrolysis by DEAD-box ATPases triggers RNA release from P-bodies and stress granules, and therefore offers an important possibility to regulate the disassembly of such bodies^12,31^.

An attractive opportunity offered by this system is the possibility to investigate the effect of biochemical activity on the material properties of the condensates since there are several mechanisms that can modulate the intrinsic ATPase activity of Dhh1. For instance, the ATPase activity can be diminished by substituting a single amino acid in the DEAD-box of the protein, resulting in the exchange of a glutamate (DEAD) to a glutamine (DQAD). This ATP hydrolysis-deficient Dhh1^DQAD^ mutant (Fig. 1a) forms constitutive processing bodies in yeast cells due to the impaired release of RNA from Dhh1, which underlies P-body dissolution. On the contrary, the ATPase activity can be stimulated by the P-body-associated factor Not1^12,46^, which leads to enhanced dynamics of P-bodies in vivo and to the dissolution of phase-separated droplets in vitro. In reconstituted systems, the hydrolyzed ATP can be regenerated, e.g., by using creatine kinase which transfers a phosphate residue on the released ADP molecule (using creatine phosphate as a donor) and the recycled ATP molecules can in turn re-promote phase separation in a cyclic way. Such “fuel”-driven turnover^47–49^ of the droplet material keeps the phase-separated system out of equilibrium and might ensure fluidity over time, preventing or delaying maturation to more solid-like, less dynamic states^31^.

Here we show that the material properties of Dhh1 condensates and their maturation over time are controlled by intrinsic features encoded in the protein sequence (LCDs) as well as extrinsic factors (ATP hydrolysis, RNA). In particular, the lack of LCDs, the presence of structured RNA, and the absence of enzymatic activity largely decrease the fluidity of the condensates, leading to their dynamic arrest.

By applying DDM we show that under most of the investigated conditions populations of condensates from the same Dhh1 sample exhibit a distribution of material properties, including subpopulations of low viscous droplets, liquid-like droplets with high viscosities, and dynamically arrested gel-/glass-like condensates.

Our results show that not only the formation of liquid–liquid phase-separated condensates but also their material properties are carefully modulated on several levels, and that biochemical activity and turnover of the droplet material increase the liquid-like properties of the condensates and prevent aging over time.

## Results

### ATP, LCDs, and RNA control the formation of the condensates

Condensates of mCherry-tagged full-length Dhh1 were formed in an aqueous buffer of 90 mM KCl, 30 mM HEPES-KOH, pH 7.4, and 2 mM MgCl_2_ in the presence of 0.5 mg/ml of the RNA analog polyuridylic acid (polyU) and 5 mM ATP/MgCl_2_ (Fig. 1). Above the threshold solubility limit at a critical concentration of *C*_*C*_ = 0.2 µM, the droplet volume increased linearly with increasing mCh-Dhh1 concentration, highly suggestive of a phase transition (Fig. 1b). In the absence of ATP and polyU, in the same buffer conditions, Dhh1 remained soluble up to around 500 µM (Supplementary Fig. 1a). We observed fusion and relaxation of adjacent droplets into larger ones, indicative of a liquid-like character of these protein-rich condensates (Fig. 1c). These results are consistent with the results of our previous analysis performed in droplet microfluidics, which shows coalescence of Dhh1 condensates over time^50^.

Analysis of the protein-rich droplets by fluorescence recovery after photobleaching (FRAP) showed an average recovery of the intensity to 61 ± 2% after 60 s (Fig. 1d).

**Fig. 2:**
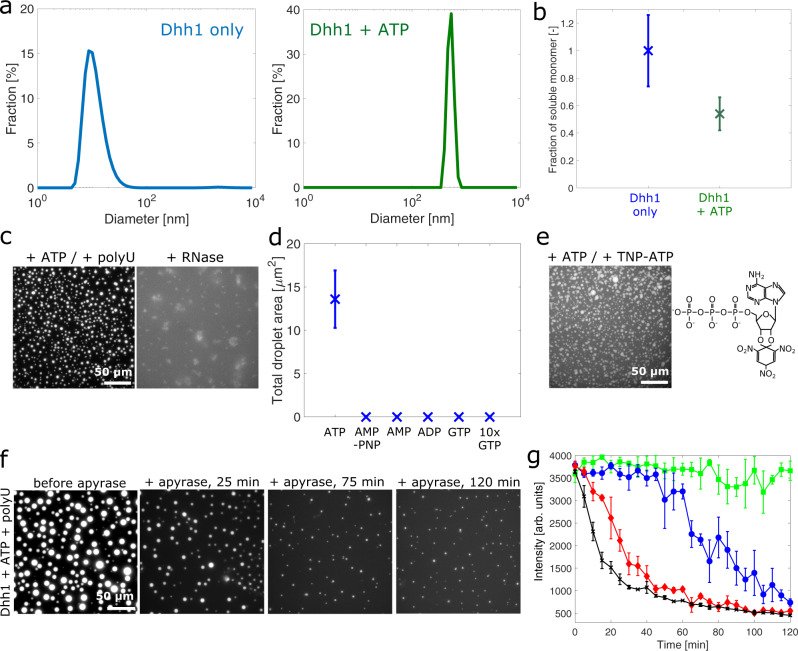
ATP and RNA control the formation and dissolution of phase-separated Dhh1-rich droplets. **a** Size distributions measured by dynamic light scattering show the formation of condensates upon the addition of 5 mM ATP to a homogenous solution of Dhh1. **b** Upon ATP binding, the fraction of soluble Dhh1 monomer decreases to 55 ± 12% with respect to a homogenous Dhh1 solution. Error bars represent the standard deviation of three different samples. **c** Dhh1 droplets formed in the presence of ATP and polyU can be dissolved by adding 36 µM RNase A, showing an additional level of regulation to form and dissolve the condensates. **d** Effect of ATP, AMP-PNP, ADP, AMP, and GTP on the phase separation of Dhh1 quantified by evaluating the mean droplet area at each condition. Error bars represent the standard deviation for >1000 individual droplets from three independent samples. **e** Fluorescence microscopy images of droplets in the presence of 2.5 mM ATP and 2.5 mM TNP-ATP, an ATP derivate that becomes fluorescent when bound to an ATP binding site. **f** Fluorescence microscopy images showing the kinetics of droplet dissolution upon addition of 1.2 µM apyrase. **g** Addition of 0 µM (green), 0.6 µM (blue), 1.2 µM (red) and 2.4 µM (black) of apyrase to droplets formed by 2 µM Dhh1 in the presence of 5 mM ATP and 0.5 mg/ml polyU concentration-dependently dissolved the droplets. The panel depicts the mean total droplet fluorescence intensity. Error bars represent the standard deviation of three different samples. Source data for panels **a**, **b**, **d**, **g** are provided in the Source Data file.

We next investigated the effects of ATP, LCDs, and polyU on the formation of the protein-rich condensates. To this aim, we analyzed an ATP-hydrolysis-deficient variant (Dhh1^DQAD^) as well as a truncated variant lacking the LCDs (Dhh1^48–425^), in addition to full-length Dhh1 (Fig. 1a). For all constructs, in the absence of ATP no LLPS was observed under the reference buffer conditions, even upon the addition of polyU (Supplementary Figs. 2–4). These results were confirmed by dynamic light scattering (DLS), which shows an average hydrodynamic diameter of 9.9 ± 3.0 nm for the full-length protein (Fig. 2a).

In contrast, the addition of an excess of 5 mM ATP/MgCl_2_ to a 5 µM Dhh1 solution resulted in the formation of non-spherical, protein particles with an average hydrodynamic diameter of 605 ± 210 nm, as analyzed by a combination of DLS and microscopy (Fig. 2a and Supplementary Fig. 2). This result, which was confirmed by the decrease in the amount of soluble monomer in presence of ATP measured by size exclusion chromatography coupled with UV absorbance (Fig. 2b), suggests an increase in intermolecular interactions upon addition of ATP. No such non-spherical particles were formed upon the addition of GTP (even up to 25 mM, Supplementary Fig. 5a), excluding an unspecific effect of the ionic strength of the nucleotide. Similar results were observed for the catalytically inactive Dhh1^DQAD^ variant (Supplementary Fig. 5b–d) and the LCD-lacking Dhh1^48–425^ construct (Supplementary Fig. 5e–g).

The addition of 0.5 mg/ml polyU to a 2 µM Dhh1 (0.17 mg/ml) solution with 5 mM ATP/MgCl_2_ acted cooperatively with ATP binding in increasing protein–protein interactions and induced the formation of spherical, liquid-like protein condensates (Figs. 1 and 2c). The formed condensates could be dissolved by degradation of polyU upon RNase A addition (Fig. 2c), demonstrating one possible mechanism to regulate the disassembly of the droplets. Dhh1^48–425^ formed smaller droplets compared to full-length Dhh1 (Supplementary Fig. 3b), likely because the presence of the LCDs increases the intermolecular interactions between adjacent Dhh1 molecules. This is consistent with previous findings showing that Dhh^48–425^ forms fewer/no P-bodies in yeast cells^31^. Furthermore, only full-length Dhh1 was able to rescue P-body formation in a yeast strain deficient in two essential P-body components (*edc3Δ lsm4ΔC*) whereas truncated Dhh1 lacking the LCDs did not^44^. In addition, the C-terminal LCD undergoes LLPS on its own in vitro (Supplementary Fig. 1b) and conjugation of the N- and C-terminal LCDs of Dhh1 to soluble proteins induces phase separation^51,52^.

We next investigated whether other nucleotides could promote phase separation of Dhh1 in the presence of polyU. To this aim, we analyzed a solution of 2 µM Dhh1 in presence of 0.1 mg/ml polyU and 5 mM of different nucleotides: ATP, its non-hydrolyzable analog adenylyl-imidophosphate (AMP-PNP), ADP, AMP or GTP (Fig. 2d and Supplementary Fig. 6). We observed the formation of condensates only with ATP. This effect is consistent with the large intramolecular rearrangements that have been shown to occur in presence of ATP, but not with ADP and AMP-PNP^43^, and indicates that these conformational changes might be crucial for the protein to be able to undergo a phase transition. Moreover, we further investigated the binding of ATP to Dhh1 inside the droplets by replacing 50% of the ATP amount with 2,4,5-trinitrophenol adenosine triphosphate (TNP-ATP), whose fluorescence intensity increases upon interactions with the ATP-binding site^53^. Fluorescence emission was detected within the protein-rich condensates (Fig. 2e), indicating that ATP is bound to Dhh1 in the condensed phase.

These results demonstrate that the ATP-bound state of Dhh1 has a high propensity to undergo phase separation, suggesting that the removal of ATP, either by hydrolysis or by dissociation from the binding site, might promote the disassembly of the protein-rich condensates. To directly test this, we introduced the enzyme apyrase, which hydrolyzes ATP with a higher reaction rate than Dhh1, into a solution of pre-formed droplets. Indeed, the addition of 0, 0.6, 1.2, and 2.4 µM apyrase induced the concentration-dependent dissolution of the droplets within 2 h (Fig. 2f, g).

Overall, our findings indicate that ATP binding increases the intermolecular protein–protein interactions of Dhh1. This mechanism is highly specific to ATP and the increase in protein–protein interactions acts synergistically with RNA binding in promoting the formation of condensates. The removal of either ATP or RNA is sufficient to dissolve the droplets providing distinct mechanisms to control the reversible assembly and disassembly of condensates.

### ATP hydrolysis, LCDs, and RNA modulate material properties and aging

**Fig. 3:**
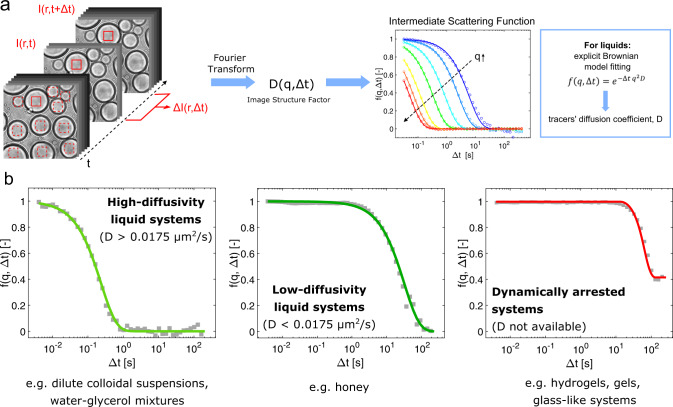
Differential dynamic microscopy to assess the material properties of biomolecular condensates. **a** Schematic representation of the DDM technique. **b** Representative intermediate scattering functions (ISF, *f*(*q*, Δ*t*)) for high-diffusivity liquid systems (water), low-diffusivity liquid systems (honey), and dynamically arrested systems (polymer-based hydrogel). Source data for panel **b** are provided in the Source Data file.

We next applied DDM to investigate how droplet activity, LCDs, and RNAs modulate the rheological properties and the maturation of the protein-rich condensates over time. DDM provides information on the dynamics of the system by analyzing a sequence of microscopy images taken in brightfield mode in time intervals of milliseconds over a time scale of seconds to minutes (Fig. 3a). In analogy to DLS, this technique reports on the sample dynamics by analyzing the fluctuations in the light scattered by the sample (see Materials and methods)^42,54^. Similar to particle-tracking strategies, the technique can be applied in combination with nano-sized particles of known size to extract diffusion coefficients. The size of the nanoparticles can be smaller than the diffraction limit as they do not have to be optically resolved in this technique. Here, we use nanotracers with a diameter of 25 nm (see Materials and methods and Supplementary Fig. 7). When Brownian motion drives the dynamics of the tracers, the correlation function provides an effective diffusion coefficient *D*.

Initially, we validated the method with mixtures of water and glycerol at different ratios and verified that the measured viscosities were consistent with values reported in the literature over a range spanning three orders of magnitude (0.001–1 Pa•s) (Supplementary Fig. 7a). Moreover, we demonstrated that DDM can be used to analyze highly viscous liquids such as honey (Fig. 3b) as well as dynamically arrested materials formed by liquid-to-gel transitions by monitoring the changes in the intermediate scattering functions (ISFs) during the gelation of the synthetic polymer polydimethylacrylamide in bulk and in micro-sized compartments generated via droplet microfluidics (Fig. 3b and Supplementary Fig. 7b, e). In the case of dynamically arrested states, diffusion coefficients cannot be computed as the particle motion in such materials is not purely diffusive.

**Fig. 4:**
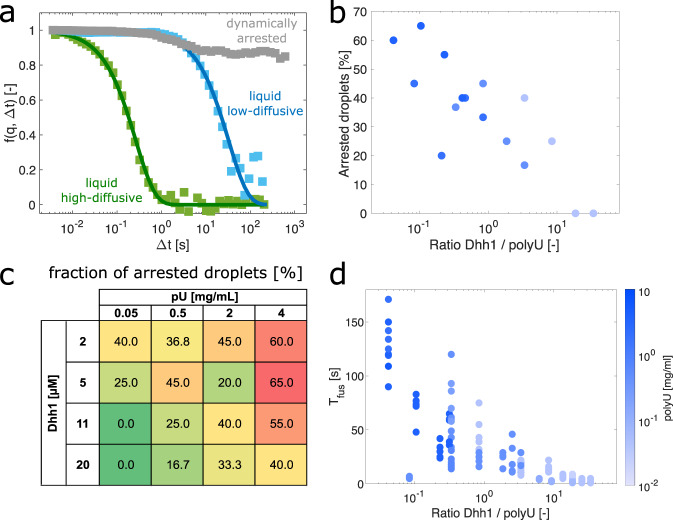
Effect of the Dhh1/polyU ratio on the droplet material properties. **a** Representative ISFs *f*(*q*, Δ*t*) of liquid-like, high-diffusive (green curve), and low-diffusive (blue curve) droplets, and dynamically arrested condensates (gray squares) observed in samples containing various Dhh1/polyU ratios. **b**–**d** Increase of the fraction of dynamically arrested droplets at low Dhh1/polyU ratios, as measured by DDM (**b**, **c**), which is consistent with the increase in the fusion time *T*_fus_ of the condensates (in **d**). In panel **c**, numbers indicate the fraction of arrested droplets in %. A high fraction of dynamically arrested droplets is highlighted in red, a low fraction is highlighted in green. The color bar in panel **d** describes the absolute polyU concentration used in panels **b**, **d**. In **b**, **c**, the fraction of arrested droplets was calculated by analyzing at least 15 different condensates in three independent samples. Source data for panels **b**–**d** are provided in the Source Data file.

After having identified polyU as an important factor to trigger the phase separation of Dhh1 (Fig. 2), we first applied DDM to investigate the effect of the Dhh1/polyU ratio on the material properties of the condensates (Fig. 4 and Supplementary Fig. 8a). We supplied the condensates with standard nanotracers with a diameter of 25 nm, whose uptake was verified by confocal microscopy (Supplementary Fig. 7c, d). In the majority of the samples, we observed different correlation functions for different condensates. Some condensates, in particular, those formed at high Dhh1/polyU ratios, exhibited a single exponential decay characteristic of liquids (Fig. 4a). Other condensates, in particular at low Dhh1/polyU ratios, exhibited DDM correlation functions which were characterized by logarithmic decays and higher plateaus (Fig. 4a), which are considered a hallmark of dynamically arrested systems such as gels and glass-like materials^39^. For each sample, we quantified the fraction of condensates characterized as dynamically arrested (Supplementary Fig. 8b).

This fraction gradually increased with decreasing the Dhh1/polyU ratio (Fig. 4b, c), consistent with the corresponding increase in the droplet fusion time *T*_fus_ (Fig. 4d). These results show that the RNA molecules that are present in P-bodies are not only clients that are processed inside these compartments but contribute to the phase separation process as well as to the modulation of material properties of the condensates.

**Fig. 5:**
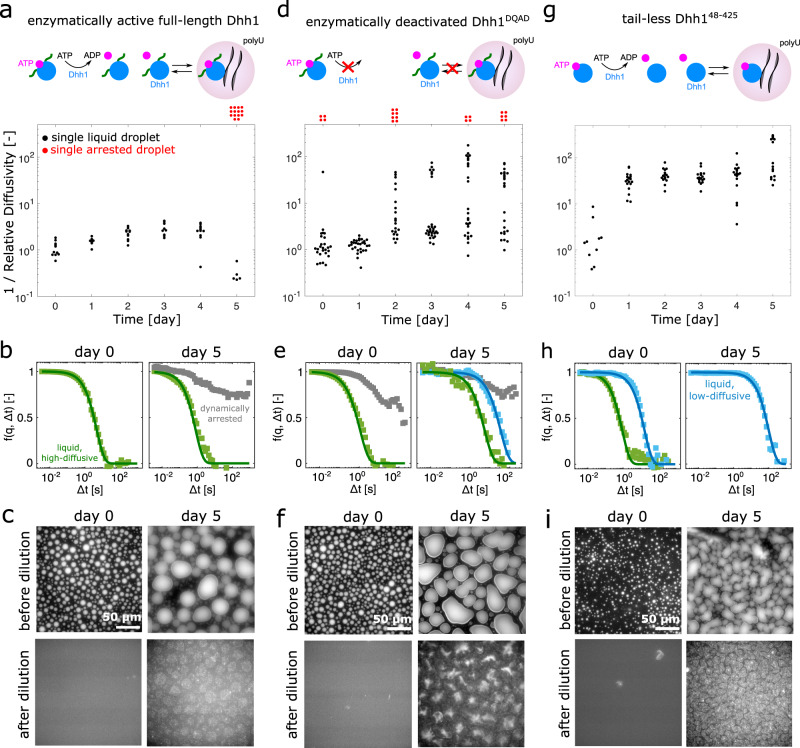
Material properties of enzymatically active full-length Dhh1, enzymatically deactivated Dhh1^DQAD^, and the LCD-lacking variant Dhh1^48–425^. The reciprocal of the relative diffusion coefficient (1 / Relative Diffusivity = 〈*D*_0_〉 / *D*(*t*)) of the condensates as well as the fraction of dynamically arrested gel-/glass-like droplets were measured over a time course of 5 days. *D*_0_ and *D*(*t*) are diffusion coefficients at time zero and time *t*, respectively. Each dot represents a single droplet. **a** Full-length Dhh1 droplets remain liquid over several days (black dots) and only a fraction of the droplets becomes dynamically arrested on day 5 (red dots). These droplets deviate from liquid-like behavior and are therefore displayed outside the graph. **b** Representative ISFs of droplets of full-length Dhh1 on day 0 (high-diffusive, green) and day 5 (high-diffusive, green; arrested, gray). **c** Droplets formed with full-length Dhh1 could be reversibly dissolved upon dilution on day 0 but not anymore on day 5, confirming the presence of non-liquid, or low-diffusive structures. **d** In addition to high-diffusive droplets, the catalytically deactivated Dhh1^DQAD^ variant showed a small fraction of dynamically arrested droplets already on day 0. The number of dynamically arrested droplets increased on day 2 and was accompanied by high- and low-diffusive droplets. **e** Typical ISFs of Dhh1^DQAD^ droplets, showing high-diffusive (green) and arrested droplets (gray) on day 0 and high-, low-diffusive (blue) and arrested droplets on day 5. **f** Also the Dhh1^DQAD^ droplets could not be dissolved by dilution on day 5, in contrast to full dissolution on day 0. **g** Droplets formed in the presence of Dhh1^48–425^ showed a decrease of the diffusivity of about one order of magnitude with respect to Dhh1 after day 1. No dynamically arrested droplets were observed over the time course of 5 days. **h** Representative ISFs of Dhh1^48–425^ droplets with high diffusivity on day 0 (green) and of droplets with low diffusivity observed after day 1 (blue). **i** Also Dhh1^48–425^ droplets could be dissolved on day 0 but not on day 5. Source data for panels **a**, **d**, **g** are provided in the Source Data file.

We next applied DDM to monitor the properties of the condensates formed by full-length, enzymatically active Dhh1, enzymatically deactivated Dhh1^DQAD^, and tail-less Dhh1^48–425^ over 5 days of incubation (Fig. 5).

Based on the previous analysis of the material properties of the droplets at various Dhh1/polyU ratios, we selected a reference condition (11 µM Dhh1, 0.05 mg/ml polyU) to observe the maturation of the condensates over time starting from a population of 100% low-viscous droplets (Fig. 5a, b). By applying the Stokes–Einstein relationship (*D* = *k*_*B*_*T/6πηR*), we estimated a viscosity in the range varying between 0.1 and 1 Pa•s, comparable to maple syrup, and only a modest change was observed during incubation (Fig. 5a). Similar viscosity values have been measured for condensates formed by proteins of the FUS family by active microrheology^36^. We note, however, that the application of the Stokes–Einstein equation has limitation for network fluids such as biomolecular condensates, since the size of the scaffold molecules can be comparable to the size of the probe. The absolute values of viscosity calculated with this approach should therefore be considered specific to the applied probes. Moreover, biomolecular condensates can possess viscoelastic properties^36,55,56^ and elastic contributions could also be present. The application of the Stokes–Einstein equation to biomolecular condensates is therefore very limited. For these reasons, we focus on the relative trends of the diffusion coefficients and of the fraction of arrested droplets as a function of time and of important biological modulators such as the architecture of the protein, the structure of RNA as well as the enzymatic activity of the protein. In the following, we report the relative changes of the diffusion coefficients (1 / Relative Diffusivity = 〈*D*_0_〉 / *D*(*t*)) over time, where *D*_0_ and *D*(*t*) are diffusion coefficients at time zero and time *t*, respectively.

In the reference sample, only on day 5 we observed the appearance of a second sub-class of condensates that exhibited arrested state (Fig. 5b). This drastic change in the rheological properties compromised the reversibility of the condensates upon dilution (Fig. 5c). These observations could be explained by the consumption of ATP over days. From previously determined hydrolysis rate (*k*_cat_ ≈ 0.001 1/s)^12,57^ we estimated that the 5 mM ATP present in the mixture would be hydrolyzed after approximately 5 days, thereby interrupting the turnover that likely keeps the droplets fluid. At this time point, condensates could be stable even in the absence of ATP probably due to conformational rearrangements of Dhh1 and polyU occurred during incubation. Consistent with this hypothesis, the condensates remain liquid after 5 days incubation when the concentration of ATP was increased to 10 mM (Supplementary Fig. 9).

It has previously been proposed that the removal of enzymatic activity could lead to hardening and irreversibility of biomolecular condensates. For instance, ATP-hydrolysis-deficient variants of Dhh1^12,58^, Ded1^59^ or DDX3X^60^, and Vasa^61^ form constitutive granules inside cells, even in the absence of stress. In contrast, ATP hydrolysis keeps these granules dynamic^31,58^.

Consistent with this hypothesis, we observed that the catalytically inactive Dhh1^DQAD^ mutant exhibited a significant fraction of condensates with ISFs corresponding to an arrested state shortly after formation (Fig. 5d, e). Moreover, already on day 2 of incubation we observed several subclasses of condensates characterized by lower diffusivity values. For this variant, the change in rheological properties also compromised the reversibility of the condensates (Fig. 5f). We note that Dhh1 and Dhh1^DQAD^ have very similar behaviors with respect to phase separation (Supplementary Figs. 2 and 4), but drastically differ in terms of their material properties.

To rule out a potential effect of the tracers, we performed experiments also in the absence of nanoparticles, analyzing the scattering signal that directly originates from the macromolecules. The shape of the autocorrelation function was consistent with and without nanoparticles, demonstrating the same qualitative behavior and the transition from liquid to an arrested state (Supplementary Fig. 10).

We next analyzed the role of the LCDs on the maturation of the droplets by investigating the behavior of the LCD-lacking variant Dhh1^48–425^ (Fig. 5g). Although these droplets showed similar phase separation behavior as full-length Dhh1 (Supplementary Fig. 3), the condensates exhibited lower values of diffusion coefficients already after 1 day of incubation, and the relative diffusivity increased by one order of magnitude over time. On day 5 we observed the presence of two subclasses of condensates, one of which exhibited remarkably low diffusion coefficients. Such values are consistent with the high viscosity of condensates formed by Laf-1, Whi3 or GAR-1ΔN, as measured by passive and active microrheology^36,62^. However, no condensates with ISFs characteristic of arrested materials were observed (Fig. 5h). Also in this case, the reversibility of the condensates upon dilution was significantly impaired on day 5 (Fig. 5i). These results show the important role of LCDs not only in modulating phase transition but also in maintaining fluid-like properties over time. This result is not intuitive since the promotion of phase separation requires attractive interactions, which however often result in high viscosity values. By contrast, the truncated variant Dhh1^48–425^ shows phase-separated droplets exhibiting higher viscosity values.

### Structured RNA induces dynamically arrested states that can be partially rescued by stimulating the turnover of the droplet material

So far, our analysis involved the RNA mimic polyU, which contains only one type of nucleotide leading to linear RNA molecules. To mimic better physiologically occurring RNAs we substituted the polyU with an in vitro-transcribed, structured, 600 nt RNA (Supplementary Fig. 11a and Materials and methods).

**Fig. 6:**
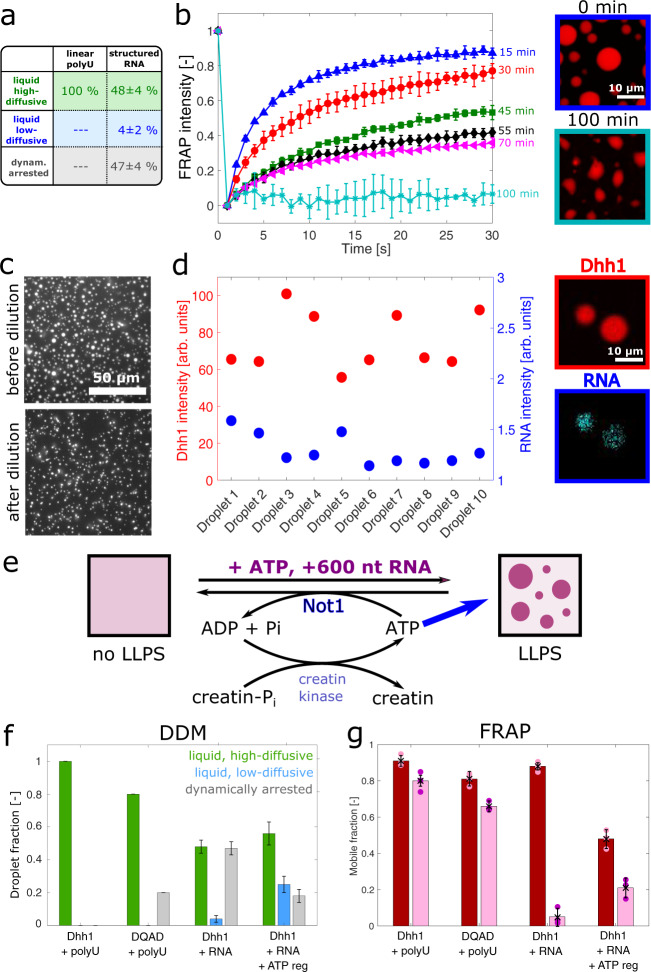
Effect of structured RNA on the dynamics of the condensates. **a** The addition of 0.05 mg/ml structured RNA (600 nucleotides) to a solution of 11 µM full-length Dhh1 and 5 mM ATP/MgCl_2_ lead to the formation of droplets with a high fraction (47 ± 4%) of glass-/gel-like droplets, in contrast with droplets formed in presence of polyU (0%). **b** Fluorescence recovery after photobleaching (FRAP) measurements showed a decrease in the mean recovery of three different droplets over a time course of 100 min. Simultaneously, the morphology of the droplets changed from an initially spherical to an irregular shape. Error bars represent the standard deviation of FRAP signals of three different droplets. **c** Condensates formed in presence of structural RNA cannot be dissolved by dilution. **d** Different droplets imaged by using confocal microscopy at the same time point exhibited different values of mCherry-tagged Dhh1 and Fluorescein-12-labeled RNA intensities. Intensity values represent the whole droplet mean intensity of individual droplets. **e** Effect of droplet turnover on material properties of biomolecular condensates. Schematic illustration of the ATP-hydrolysis-regeneration system. **f** Fractions of the different droplet subpopulations characterized by DDM over 100 min of incubation: high-diffusive liquid (green), low-diffusive liquid (blue), and dynamically arrested (gray). Error bars represent the standard error of the mean of at least 15 different droplets per condition. **g** Mean mobile fraction extracted from FRAP measurements at time 0 (dark red) and after 100 min incubation (light red). In presence of polyU, the mobile fraction was about 91 ± 3% and remains almost constant over time. A similar behavior was observed for the Dhh1^DQAD^ variant. When polyU was replaced with structured RNA, the mobile fraction decreased to 5 ± 5% over time, and this decrease could be partially rescued when coupled to an active system. Error bars represent standard deviation of mobile fractions of three different droplets. Source data for panels **b**, **d**, **f**, **g** are provided in the Source Data file.

Upon addition of this structured RNA, Dhh1 again formed condensates instantaneously after mixing (Supplementary Fig. 11b). Analysis of the material properties by DDM revealed that these droplets contained a large subpopulation of dynamically arrested droplets (high-diffusive, 48 ± 4%; low-diffusive 4 ± 2%; and dynamically arrested, 47 ± 4%) compared to the system containing polyU (low-viscous, 100%) (Figs. 5b and 6a), even directly after formation. This loss of droplet fluidity in presence of structured RNA was confirmed by FRAP experiments, which showed that the fraction of mobile molecules (defined as the percentage of recovery after 30 s) was about 88 ± 2% directly after formation (Fig. 6b) and decreased rapidly to around 5 ± 5% after 100 min. Droplets formed at the same concentration of polyU showed higher recovery after the same time (Supplementary Fig. 11c). Furthermore, in the presence of structured RNA, the condensates underwent changes in morphology from a spherical to an irregular shape (Fig. 6b). In addition, these condensates could not be dissolved by dilution, confirming their non-liquid nature (Fig. 6c).

The heterogeneity of droplet material properties could occur due to a non-homogeneous distribution of Dhh1 and RNA molecules among the condensates. Consistent with this hypothesis, measurements of the fluorescence intensities of mCherry-tagged Dhh1 and Fluorescein-12-labeled RNA within ten different condensates (Fig. 6d and Supplementary Fig. 11d) showed differences of almost 40% in the protein signal and of 30% in the RNA signal. This heterogeneity in composition indicates low dynamics of material exchange between the different condensates after their formation, suggesting that stronger protein–protein, RNA–RNA, and protein-RNA interactions lead to the formation of heterogeneous networked fluids.

We next attempted to restore droplet dynamicity also in presence of structured RNAs by promoting the turnover of the droplet-forming/droplet-dissolving material.

To this aim, we stimulated the ATPase activity of Dhh1 by adding Not1 into the system, which induces droplet dissolution^12^. Simultaneously, we converted the resulting ADP into ATP by the addition of creatine kinase (using creatine phosphate as phosphate donor). Using this active system, we promote a rapid exchange of proteins and RNA molecules between the condensed and diluted phase, until all creatine phosphate (our “fuel”) was consumed (Fig. 6e). Under these conditions, the arrested droplets could partially be rescued and the average number of droplets with arrested dynamics was significantly decreased to about 18 ± 4%, which corresponds to a reduction of 30% compared to the non-active system (Fig. 6f). These findings were consistent with the higher mobile fraction compared to the non-active system measured by FRAP after 100 min (Fig. 6g).

These results show that biomolecular condensates formed via active phase-separated systems that are constantly turned over by biochemical reactions remain fluid over longer time scales, indicating that the coupling of biochemical reactions with phase separation provides a mechanism to prevent or at least delay droplet maturation.

## Discussion

Here, we have investigated mechanisms controlling the material properties of biomolecular condensates consisting of the DEAD-box ATPase Dhh1, a simplified in vitro model of P-bodies. To this aim, we introduced DDM, which allowed us to probe the dynamics of the condensates and distinguish between liquid-like and arrested states. The DDM analysis can be performed in situ, even in the absence of tracer particles and at the single condensate level. The latter aspect was very important in revealing that Dhh1 condensates exhibit a distribution of material properties that can be modulated by various factors.

To investigate the molecular basis underlying this rich behavior, we first characterized the effect of multiple factors (LCDs, ATP and RNA) on the reversible formation and dissolution of the droplets (Figs. 1 and 2). We observed a hierarchy of intermolecular interactions^63^ encoded by these different modulators. Based on the emerging “stickers and spacers” model^64^, the molecular architecture of the interacting biomolecules can be divided into stickers that mediate the intermolecular interactions driving LLPS and spacers that modulate other chain properties. In the absence of RNA, we propose that the LCDs of Dhh1 act as stickers mediating weak multivalent interactions, while the globular RecA domains represent spacers. Indeed, the C-terminal LCD of Dhh1 undergoes LLPS on its own (Supplementary Fig. 1b). In the absence of RNA, we observed phase separation only at low salt concentrations, which could strengthen attractive LCD–LCD interactions. However, in presence of RNA, the RNA–protein interactions dominate over the weak multivalent interactions of the LCDs and therefore these heterotypic interactions become the sticky element that determines the phase behavior. These stronger RNA–protein interactions are less sensitive to changes in the salt concentration (Supplementary Fig. 2).

Surprisingly, the DDM analysis showed that in case of Dhh1, the LCDs do not only promote LLPS, but they are also important in maintaining droplet fluidity over time. Indeed, droplets formed by the LCD-lacking variant Dhh1^48–425^ exhibited a decrease in diffusivity over days than droplets formed in presence of full-length Dhh1. This suggests that the LCDs may switch from being a driver of phase separation in absence of polyU to being a modulator of the material properties in presence of polyU. We propose that this is an additional important function of LCDs in biomolecular condensates (Fig. 5), consistent with recent observations on droplets formed by the Dhh1 homolog Me31B in Drosophila oocytes^65^.

Furthermore, we identified the structure of RNA as another important external modulator of the material properties of the condensates. Specifically, structured RNA drastically decreased the dynamics of a large subpopulation of the Dhh1 condensates. The presence of structured RNA in these condensates increases the complexity of the system. RNA base pairing leads not only to a complex three-dimensional structure of the RNA itself, but allows also for intermolecular RNA–RNA interactions, which are further promoted by the high RNA concentration inside the droplets. While some of these interactions could be transient and dynamic, others could be energetically favored and stabilized over time, and might thereby contribute to the formation of networked droplets and droplet hardening.

Importantly, we find droplet subpopulations characterized by different material properties. This highlights the importance of single-droplet techniques to analyze these heterogeneous populations. This broad distribution of material properties can originate from differences in the protein/RNA concentration inside the droplets (Fig. 6), which in this non-equilibrium system can be further amplified by changes over time (aging).

We have further demonstrated that droplet activity is an important mechanism to modulate their material properties. In this work, we refer to “active droplets” as the formation and degradation of our condensates depending on biochemical reactions that simultaneously generate components characterized by low and high phase separation propensities^47,48,66,67^ (Fig. 6e).

The Dhh1 system allows us to investigate the effect of activity on the droplet material properties on several levels since the intrinsic enzymatic ATPase activity of Dhh1 can be modulated in different ways.

Condensates formed in presence of full-length Dhh1 and polyU exhibit high fluidity, despite the low intrinsic propensity to hydrolyze ATP^12^. We note that under these conditions the hydrolyzed ATP is not actively regenerated, and our results suggest that ATP consumption is therefore the limiting factor determining the dynamic arrest of the droplets on day 5 (Fig. 5a).

Condensate fluidity can be decreased when ATP hydrolysis is inhibited by exchanging a single glutamate (E) to glutamine (Q) in the DEAD box of the protein (Dhh1^DQAD^)^12,58^. Droplets formed in presence of this variant rapidly form large populations of low-diffusive and gel-/glass-like droplets showing the importance of enzymatic activity in their interior (Fig. 5d).

While condensates formed in presence of full-length Dhh1 and polyU exhibit highly liquid properties, condensates formed in presence of structured RNA are largely dynamically arrested, even shortly after their formation. Increasing the Dhh1 activity employing an ATP hydrolysis-regeneration system (Fig. 6e) promotes liquidity and partially rescues the dynamic arrest of the droplets (Fig. 6f). This is likely due to the accelerated turnover of the droplet material between the dispersed and the diluted phase, which keeps the system out of equilibrium. However, this increase in droplet turnover was not sufficient to fully liquefy all droplets, indicating the importance of structured RNA in tuning the material properties of condensates and in being a critical driver for their dynamic arrest (Fig. 6f).

Since P-bodies are part of the cellular metabolism and are therefore intrinsically out-of-equilibrium systems, this role of biochemical reactions in preserving fluid-like properties can be important also in the cellular context to maintain functional P-bodies and counteract the effect of client mRNAs, which may otherwise compromise their dynamics.

Furthermore, our study shows that liquid-like condensates can “age” toward dynamically arrested materials over time, consistent with recent findings demonstrating that the relaxation time of some condensates increases with age, in analogy with a glass-forming system^36^.

These results show that biomolecular condensates are carefully regulated on several levels by nature not only to reversibly assemble and disassemble in the presence of suitable triggers but also to maintain the appropriate level of fluidity required for their function. Some of the regulating features are embedded in the architecture of the scaffold components, such as the presence of LCDs in the protein or the structure of the RNA molecules.

**Fig. 7:**
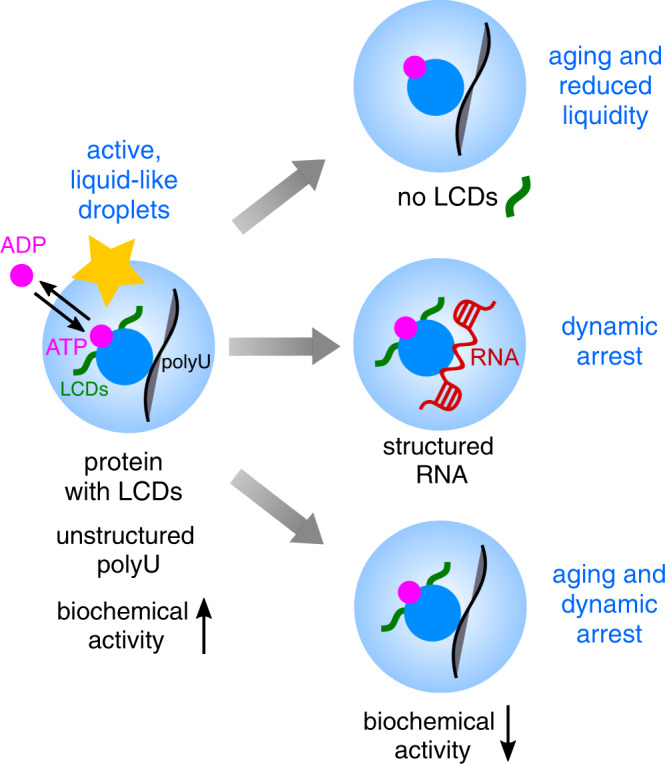
The low-complexity domains, the RNA structure, and the presence of biochemical reactions/droplet turnover affect the material properties and the aging of liquid–liquid phase-separated droplets. Droplet material properties are modulated by the droplet composition: the presence of LCDs and unstructured polyU results in liquid-like Dhh1 droplets, while the lack of LCDs and structured RNA lead to large fractions of dynamically arrested droplets. Droplets formed in the presence of enzymatically active DEAD-box ATPase Dhh1 remain liquid-like over days while the suppression of the enzymatic activity of Dhh1 leads to dynamic arrest and droplet solidification.

In particular, we have shown that biochemical activity and turnover of the droplet material increase the liquid-like properties of the condensates and prevent aging over time (Fig. 7).

## Methods

### Protein expression and purification

Expression and purification of mCherry-tagged Dhh1, Dhh1^48–425^, Dhh1^DQAD^, and non-tagged MIF4G-Not1 was performed as previously described^12^. Briefly, competent *Escherichia coli* BL21-Gold (DE3) strains were transformed via heat shock at 42 °C with plasmids carrying the genes for Dhh1 (pKW3631), Dhh1^48–425^ (pKW4063), Dhh1^DQAD^ (pKW3632) or Not1^MIF4G^ (pKW3469). Each plasmid was carrying sequences for a 6× His tag and ampicillin resistance. Cells were cultured in LB medium at 37 °C and protein expression was induced by the addition of 0.5 mM (0.2 mM for Not1^MIF4G^) isopropyl-beta-D-1-thiogalactopyranoside. After harvesting, cells were resuspended in lysis buffer (pH 7.5, 300 mM NaCl, 50 mM Tris, 10 mM imidazole, 10% glycerol) and lysed by sonication. Protein purification was performed via affinity chromatography using Ni^2+^ charged Fast Flow Chelating Sepharose (GE Healthcare) as a stationary phase. This step was followed by size exclusion chromatography on a Superdex 75 column (GE Healthcare) using a solution at pH 7.5, 300 mM NaCl, 25 mM Tris, 2 mM 2-Mercaptoethanol, and 10% glycerol as elution buffer. Purified fractions were pooled, concentrated, and flash-frozen in liquid nitrogen. The phase diagram of Dhh1 was typically analyzed in 30 mM HEPES-KOH buffer at pH 7.4 supplied with 150 mM KCl and 2 mM MgCl_2_.

### In vitro transcription and labeling of RNA

For in vitro transcription, a construct was designed consisting of a 6 × 100 nucleotide repeat of actin mRNA interspaced by 6 different restriction site linkers (Supplementary Fig. 11a) and commercially synthesized (GeneWiz). After amplification in *E. coli* bacteria and isolation from the cells using a QIAprep Spin Miniprep Kit (Qiagen), the plasmid was linearized by restriction enzyme digestion at the last restriction site RS6 (Supplementary Fig. 11a), by adding 1 µl (20 units) of BamHI restriction enzyme (New England Biolabs) to 1 µg DNA. The linearized DNA was purified on a 1% agarose gel, isolated from the gel, and in vitro transcribed using a MEGAshortscript^TM^ Transcription Kit (ThermoFisher Scientific) according to the manufacturer’s instructions. For labeling, 0.9 mM of Fluorescein-12-labeled UTP (Jena Bioscience, Germany) was added. The mixture was incubated overnight at 37 °C and the synthesized RNA was purified by ethanol precipitation.

### Sample preparation and microscopy

For in vitro droplet formation, buffer, ATP, and RNA were premixed on ice and transferred into a 384-well plate (Brooks, Matriplate). To induce the phase separation, a homogeneous protein stock solution was added to the mixture, resulting in a total volume of 20 µl. Samples were imaged after the droplets had settled at the bottom of the plate, by either widefield microscopy or confocal fluorescence microscopy. Analysis by widefield microscopy was performed on an inverted epi-fluorescence microscope (Nikon Eclipse Ti-E; MicroManager software, version 2.0 gamma) equipped with a 60x NA 1.4 oil objective (Nikon), an LED light source (Omicron LedHUB Light engine; Omicron Software, version 3.9.28) and an Andor Zyla sCMOS camera. Confocal microscopy images were acquired with an inverted epi-fluorescence microscope (Leica TCS SP8; Leica Application Suite X (LAS X) software, version 1.0) equipped with a 63× NA 1.4 oil objective (Leica), a Laser unit for confocal acquisition (AOBS system) and a sCMOS camera (Hamamatsu Orca Flash 4.0).

The size distributions of the droplets were reconstructed from the images acquired by optical and fluorescence microscopy via an in-house program written in Matlab (version R2020a). Fluorescence intensities of the droplet and soluble phase were extracted using a different in-house written Matlab program. To define the condensate and the background, an arbitrary intensity threshold was set to define pixels with high intensities (droplets) and low intensities (soluble phase).

### Soluble monomer concentration

The soluble Dhh1 concentration (C_S_) was measured by UV absorbance after removing the protein-rich droplets by centrifugation (10 min at maximum speed) on a bench top centrifuge and running the supernatant on a Superdex 200 size exclusion column (GE Healthcare) connected to a high-pressure liquid chromatography system (1100 series, Agilent), by controlling the flow using the OpenLab ChemStation software (version 0.0.1.98).

### Dynamic light scattering

We used DLS to measure the hydrodynamic radius of the protein and the resulting condensates. 100 µl of the samples were prepared in a quartz cuvette (Hellma Analytics, Germany) and measured on a Zetasizer Nano ZS instrument (Malvern; Zetasizer Nano software, version 7.13) working in 173° backscattering mode.

### Fluorescence recovery after photobleaching (FRAP)

FRAP experiments were performed on the confocal microscope described above. Droplets were bleached by focusing a 561 nm laser light on a circular area with a diameter equal to about one-tenth of the total droplet diameter. Image analysis, including background subtraction, correction of bleaching during recovery, and normalization to pre- and postbleach intensity was performed via an in-house program written in Matlab.

### Differential dynamic microscopy (DDM)

Within a microscopy image, the intensity value corresponding to a pixel located in position (*x, y*) at time *t* is denoted with *I*(*x, y;t*). Fluctuations in the values of *I*(*x,y;t*) can be induced by the motion of colloidal particles employed as tracers, or alternatively—in the absence of tracers—by fluctuations in the intrinsic refractive index of the sample caused by rearrangements of structural sub-regions. Such fluctuations can be measured by DDM and give important information about the dynamics of the sample of interest. A spatial variance of the type

1 $${\sigma }^{2}\left(\Delta t\right)=\mathop{\sum}\limits _{x}\mathop{\sum}\limits _{y}{\left|D(x,y{{{{{\rm{;}}}}}}\Delta t)\right|}^{2}=\mathop{\sum}\limits _{x}\mathop{\sum}\limits _{y}{\left|I\left(x,y{{{{{\rm{;}}}}}}t+\Delta t\right)-I(x,y{{{{{\rm{;}}}}}}t)\right|}^{2}$$

can thus be defined for images that are Δ*t* apart in time (Eq. 1). The time-averaged Fourier transform of *D*(*x,y;*Δ*t*), denoted with ${\mathfrak{D}}\left({{{{{\boldsymbol{q}}}}}};\Delta t\right)={\left\langle {{{{{\mathcal{F}}}}}}\{D(x,{y;}\Delta t)\}\right\rangle }_{t}$, can be further radially averaged for isotropic samples, leading to the loss of the dependence on the orientation of the wavevector ***q*** and yielding a simpler ${\mathfrak{D}}({q;}\Delta t)$^42,68^. It can be shown that the relation:

2 $${\mathfrak{D}}\left(q{{{{{\rm{;}}}}}}\Delta t\right)=A\left(q\right)\left[1-f(q,\triangle t)\right]+B(q)$$

holds, where *A*(*q*) is related to the Fourier transform of the microscope Point Spread Function, *B*(*q*) accounts for the camera noise, and $f(q,\triangle t)$ is the ISF of the system that can be traditionally determined by DLS measurements (Eq. 2).

If an explicit analytical model for the ISF is known, as in the case of tracers that undergo Brownian diffusion in a Newtonian fluid, a straightforward determination of the rheological properties of the sample is possible by direct fitting of ${\mathfrak{D}}\left({q;}\Delta t\right)$^42,68,69^.

To perform DDM experiments, a sample volume of 20 µl was introduced in at least three wells of a 384-well plate with quartz bottom (Matriplate, Brooks Life Sciences, USA). In all, 4 µl of fluorescently labeled nanotracers with a diameter of 25 nm (micromer®-greenF, Micromod Partikeltechnologie GmbH, Rostock, Germany) were added to each well. The samples were stored at 4 °C over several days and equilibrated to room temperature before the measurement.

Stacks of brightfield images were acquired on a Ti2-U epi-fluorescence inverted microscope (Nikon) equipped with an sCMOS camera (Zyla 4.2P-CL10, Andor, UK) and with a ×60 magnification water objective (CFI Apochromat NIR 60X W, Nikon, Japan, NA = 1.0). Sequences of *N* = 1000–4000 images of 512 × 512 pixels (corresponding to 55.3 × 55.3 µm^2^) were acquired both at high frequency (250 frames per second (fps)) and low frequency (4 fps) to capture the short- and long-time sample dynamics, respectively. The exposure time was kept fixed throughout the experiments at 1 ms. The images were processed and analyzed with a custom-written Matlab code based on ref. ^68^. The size of the selected regions of interest (ROIs) varied between 64 × 64 and 256 × 256 pixels, depending on the samples. The chosen ROIs were smaller than the probed condensate to exclude signal from the droplet surface. Moreover, a Blackman–Harris window was applied during the analysis to increase the weight of the signal in the droplet center with respect to the edges.

### Statistics and reproducibility

All micrographs in this study are representative images of experiments carried out with at least three repetitions.

### Reporting summary

Further information on research design is available in the Nature Research Reporting Summary linked to this article.

## Supplementary information

Supplementary Information

Reporting Summary

## Data Availability

The data supporting the findings of this study are available from the corresponding authors upon reasonable request. Source data for the figures and supplementary figures are provided as a source data file. Source Data are provided with this paper.

## Source data

Source Data

## Code availability

For DDM analysis we adapted a previously published Matlab code^68^ (GitHub, 10.5281/zenodo.30559). Adapted codes and all other used codes for data analysis are available from the corresponding authors upon request.
