# Supplementary material for: Dynamic arrest and aging of biomolecular condensates are modulated by low-complexity domains, RNA and biochemical activity
Source: Nat Commun. 2022 May 31;13:3030. doi: 10.1038/s41467-022-30521-2 (PMC9156751; doi:10.1038/s41467-022-30521-2)
Supplement: Supplementary file 1 — Supplementary Information [file 41467_2022_30521_MOESM1_ESM.pdf]

Supplementary Information:

Dynamic arrest and aging of biomolecular condensates are modulated by low-complexity domains, RNA and biochemical activity

Miriam Linsenmeier[1], Maria Hondele[2,3], Fulvio Grigolato[1], Eleonora Secchi[4], Karsten Weis[2,*], Paolo Arosio[1,*]

[1]*Department of Chemistry and Applied Biosciences, Institute for Chemical and Bioengineering, Swiss Federal Institute of Technology, 8093, Zurich, Switzerland*

[2]*Department of Biology, Institute for Biochemistry, Swiss Federal Institute of Technology, 8093, Zurich, Switzerland*

[3]*Biozentrum, Center for Molecular Life Sciences, University of Basel, 4056, Basel, Switzerland*

[4]*Department of Civil, Environmental and Geomatic Engineering, Swiss Federal Institute of Technology, 8093, Zurich, Switzerland*

**To whom correspondence should be addressed:*
paolo.arosio@chem.ethz.ch; karsten.weis@bc.biol.ethz.ch

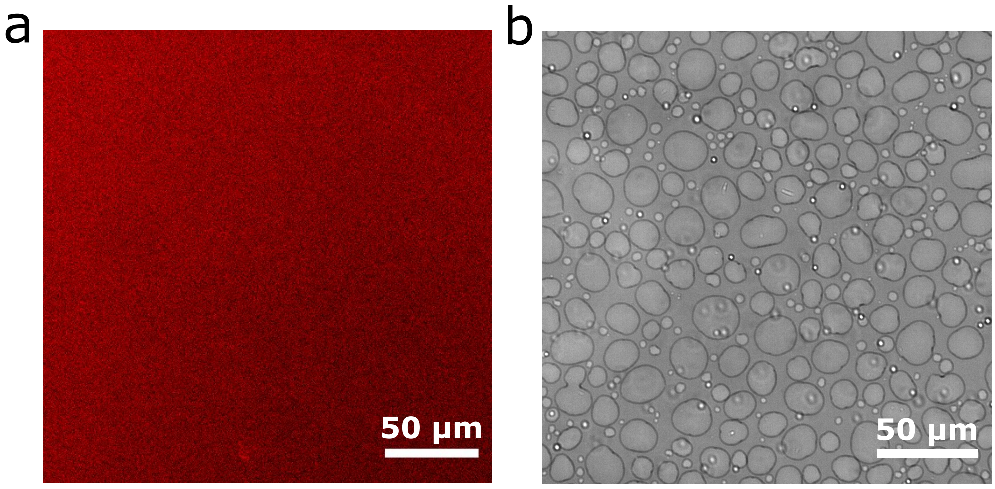

Suppl. Fig. 1: a 500 µM Dhh1 solution does not undergo phase separation in absence of ATP and RNA. **b** Droplets formed by the C-terminal low-complexity domain of Dhh1 in the presence of 50 mM Tris, pH 7.0, 50 mM NaCl.

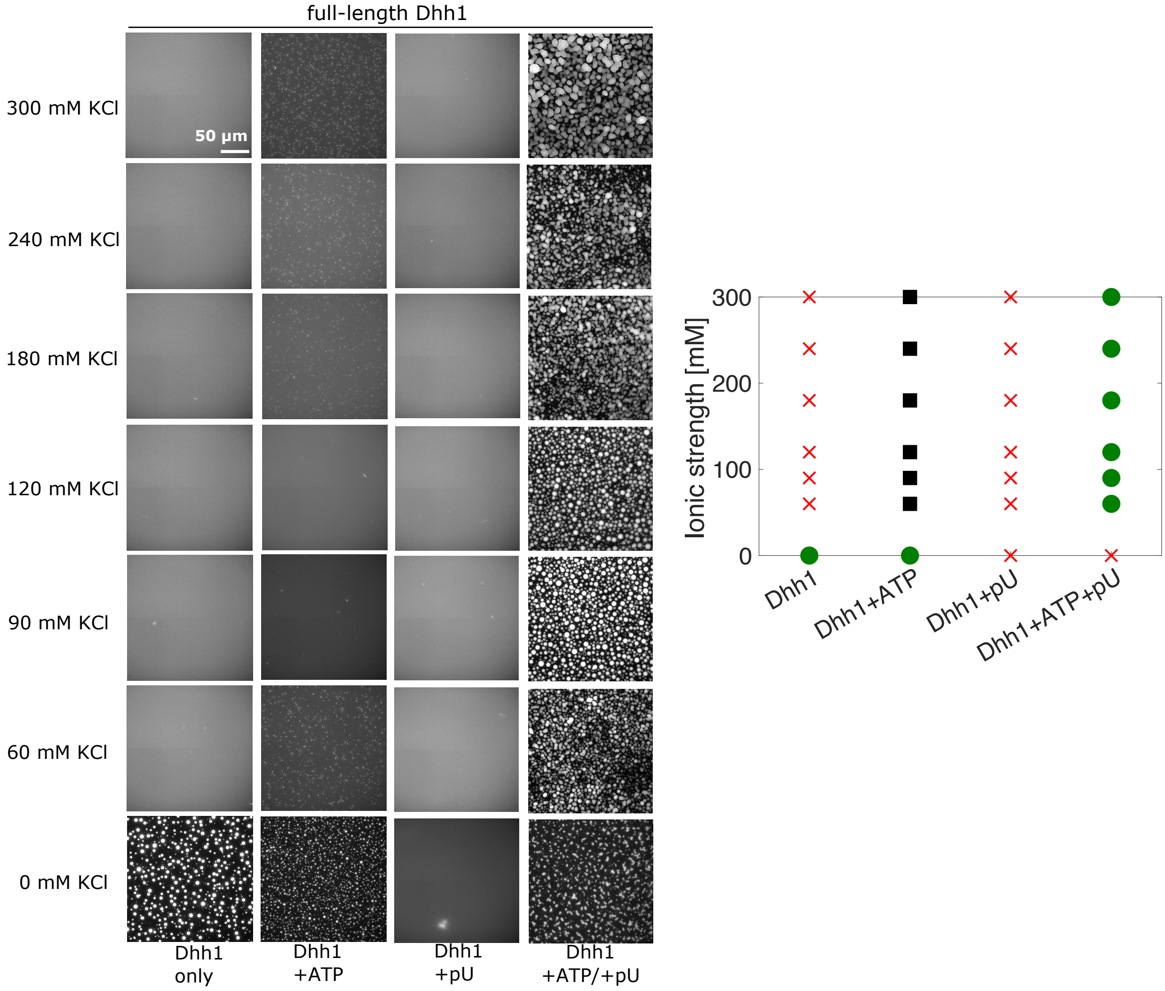

Suppl. Fig. 2: Phase diagram of full-length Dhh1 in absence / presence of ATP, polyU and ATP/polyU at increasing KCl concentration. Left: fluorescence microscopy images; right: phase diagram. Red crosses – no phase separation, green circles – phase separation, black squares – small aggregates.

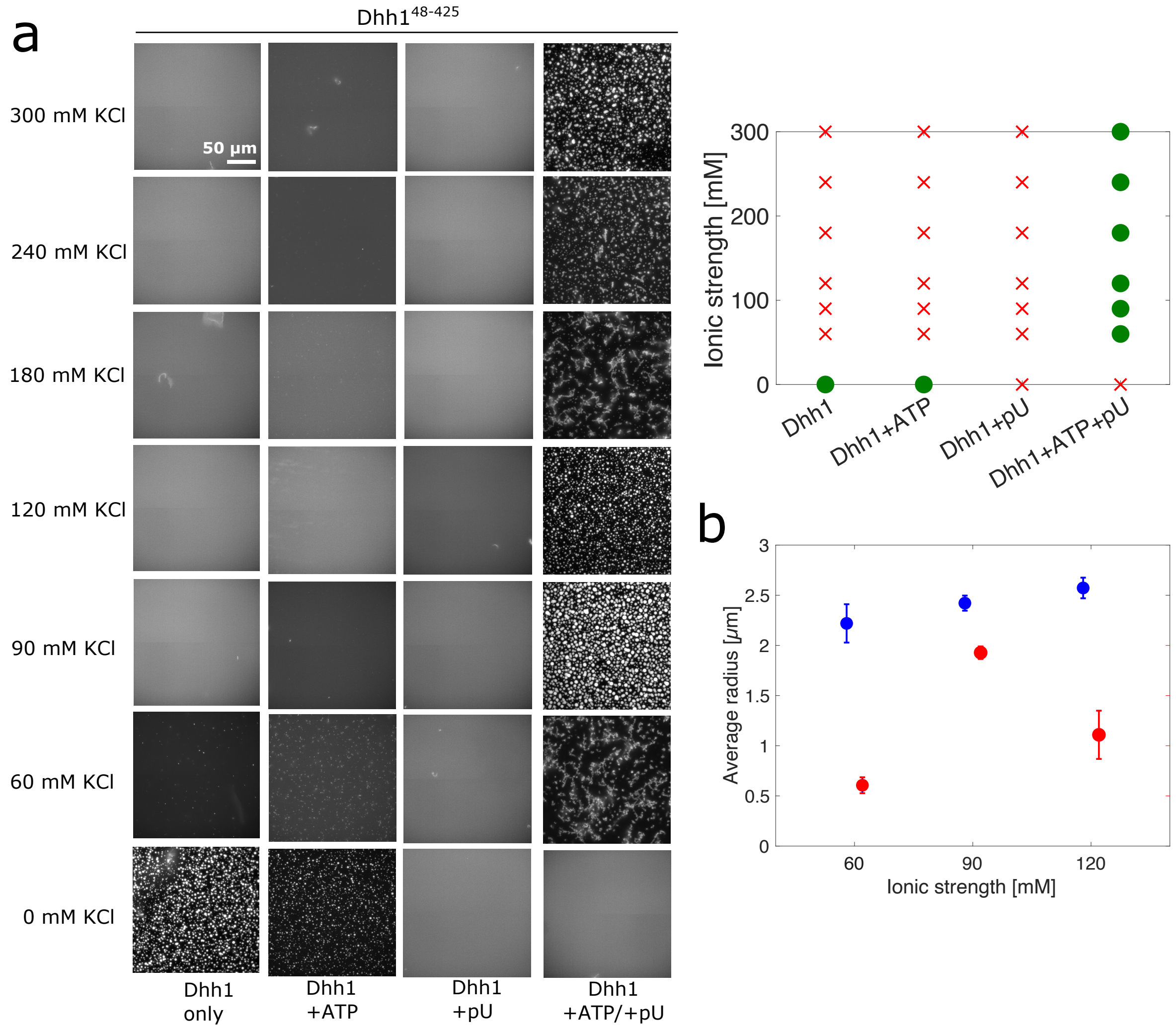

Suppl. Fig. 3: Phase diagram of Dhh1$^{48\text{-}425}$, lacking the N- and C-terminal low-complexity domains in absence / presence of ATP, polyU and ATP/polyU at increasing KCl concentration. **a** Left: fluorescence microscopy images; right: corresponding phase diagram. Red crosses – no phase separation, green circles – phase separation, black squares – small aggregates. **b** Comparison of the mean average droplet size of full-length Dhh1 and Dhh1$^{48\text{-}425}$ at 60, 90 and 120 mM KCl. In all cases, the droplets formed in presence of Dhh1$^{48\text{-}425}$ (red crosses) are smaller than in presence of full-length Dhh1 (blue crosses). Error bars represent the standard deviation of the droplet size of >2000 droplets from 3 independent samples. Source data of panel (b) are provided in the Source Data file.

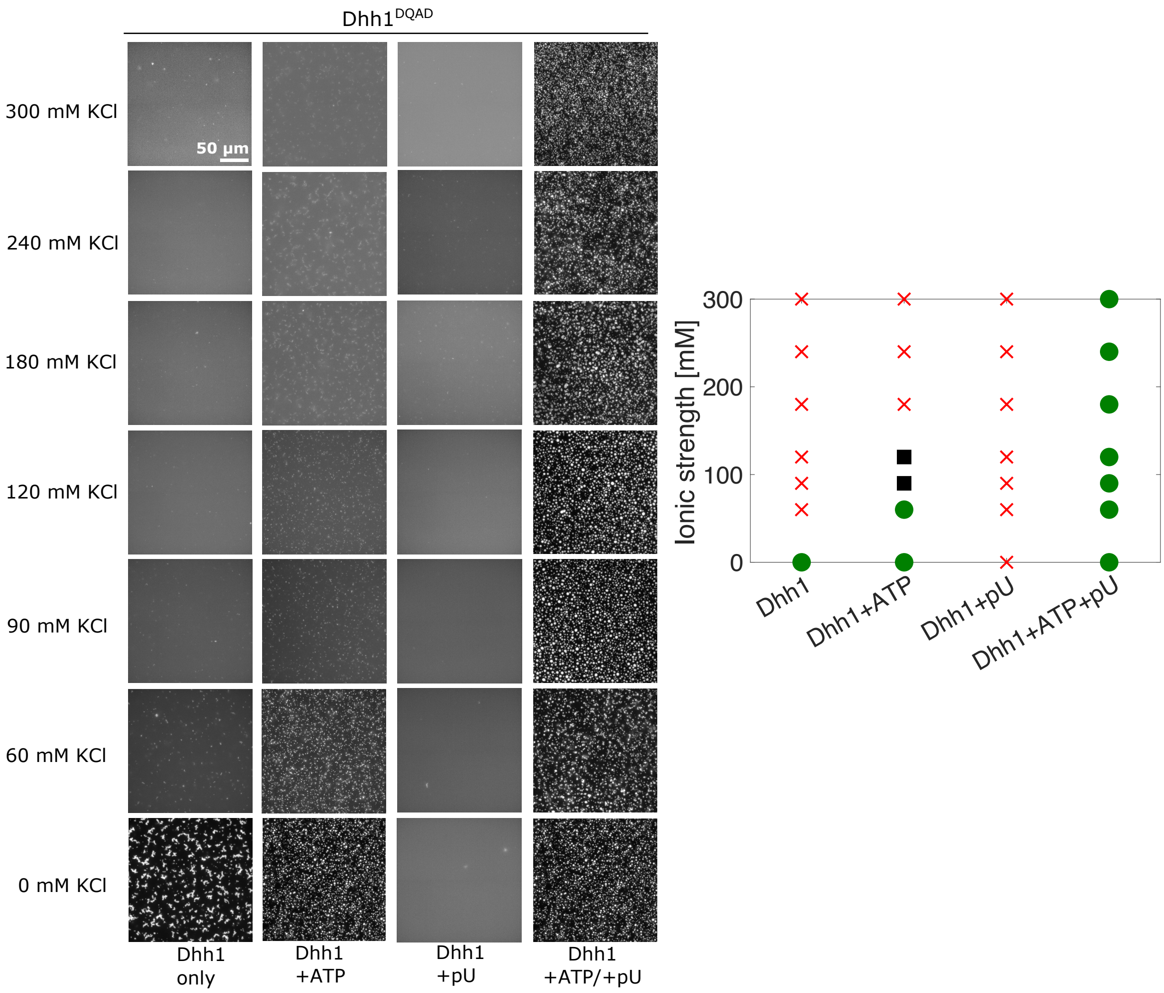

Suppl. Fig. 4: Phase diagram of Dhh1^{DQAD} deficient in ATP hydrolysis propensity in absence / presence of ATP, polyU and ATP/polyU at increasing KCl concentrations. Left: fluorescence microscopy images; right: corresponding phase diagram. Red crosses – no phase separation, green circles – phase separation, black squares – small aggregates.

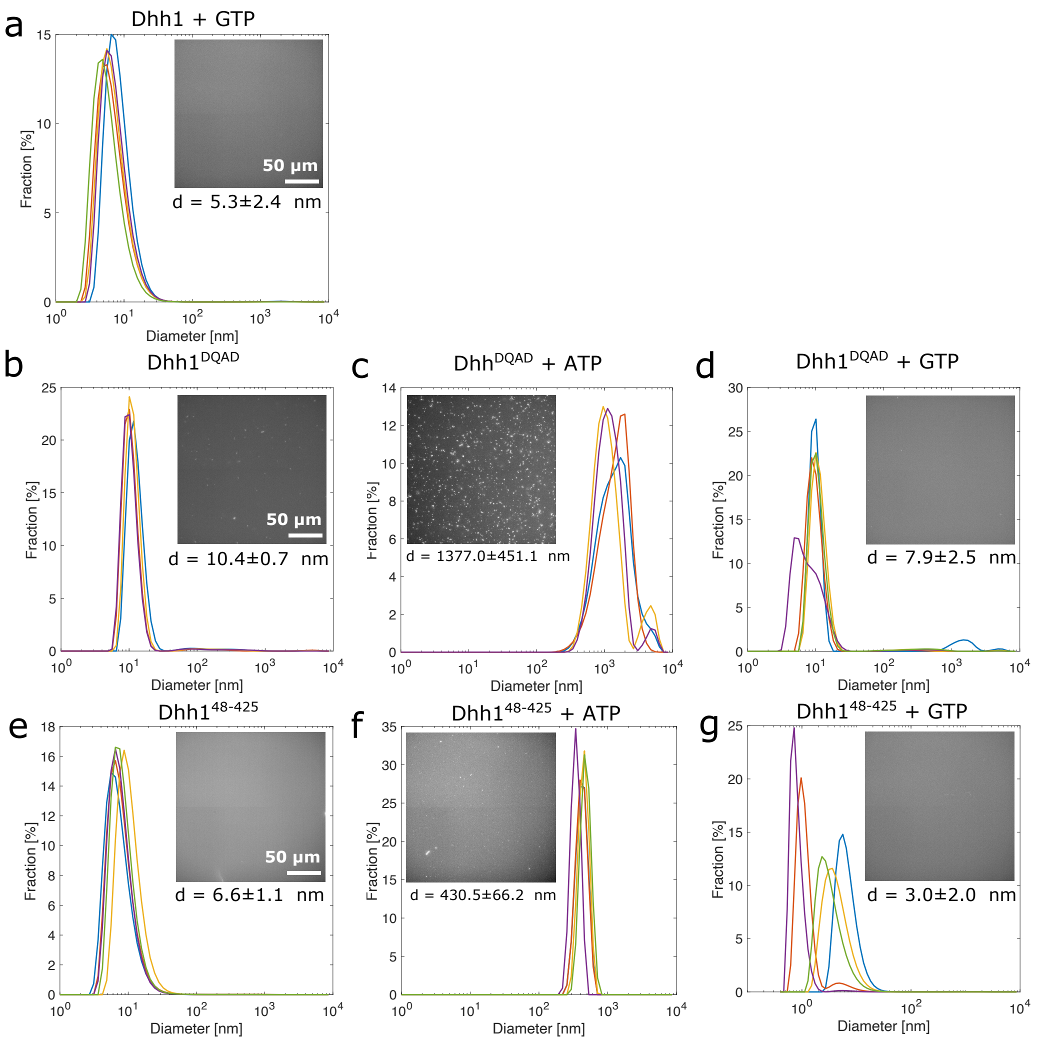

Suppl. Fig. 5: Effect of ATP and GTP on the phase transition propensity of full-length Dhh1, Dhh1$^{48\text{-}425}$, Dhh1^{DQAD} measured by dynamic light scattering and fluorescence microscopy **a** Full-length Dhh1 remains in its monomeric form in presence of 25 mM GTP with an average diameter of 5.3 ± 2.4 nm. **b-d** Also Dhh1^{DQAD} remains soluble in absence of ATP and GTP, as well as in presence of 25 mM GTP, but forms large condensates in presence of 5 mM ATP. **e-g** Dhh1$^{48\text{-}425}$ remains soluble in absence of ATP and GTP (a) and in presence of 25 mM GTP (c). Only the addition of 5 mM ATP induced the formation of small condensates with an average diameter of 430.5 ± 66.2 nm. Source data of panels (a-g) are provided in the Source Data file.

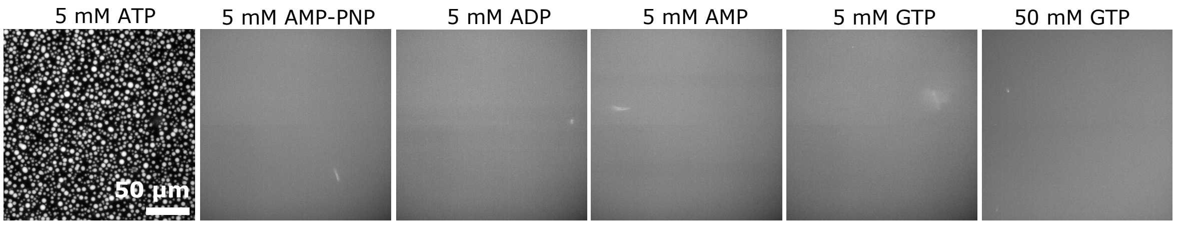

Suppl. Fig. 6: Specificity of various nucleotides to induce phase separation. Only in presence of 5 mM ATP droplet formation was induced. In presence of 5 mM non-hydrolysable AMP-PNP, ADP, AMP and GTP the full-length Dhh1 solution (including 0.5 mg/ml polyU) remained soluble. Even the addition of 50 mM GTP did not induce droplet formation, excluding effects of the ionic strength. Source data of this figure are provided in the Source Data file.

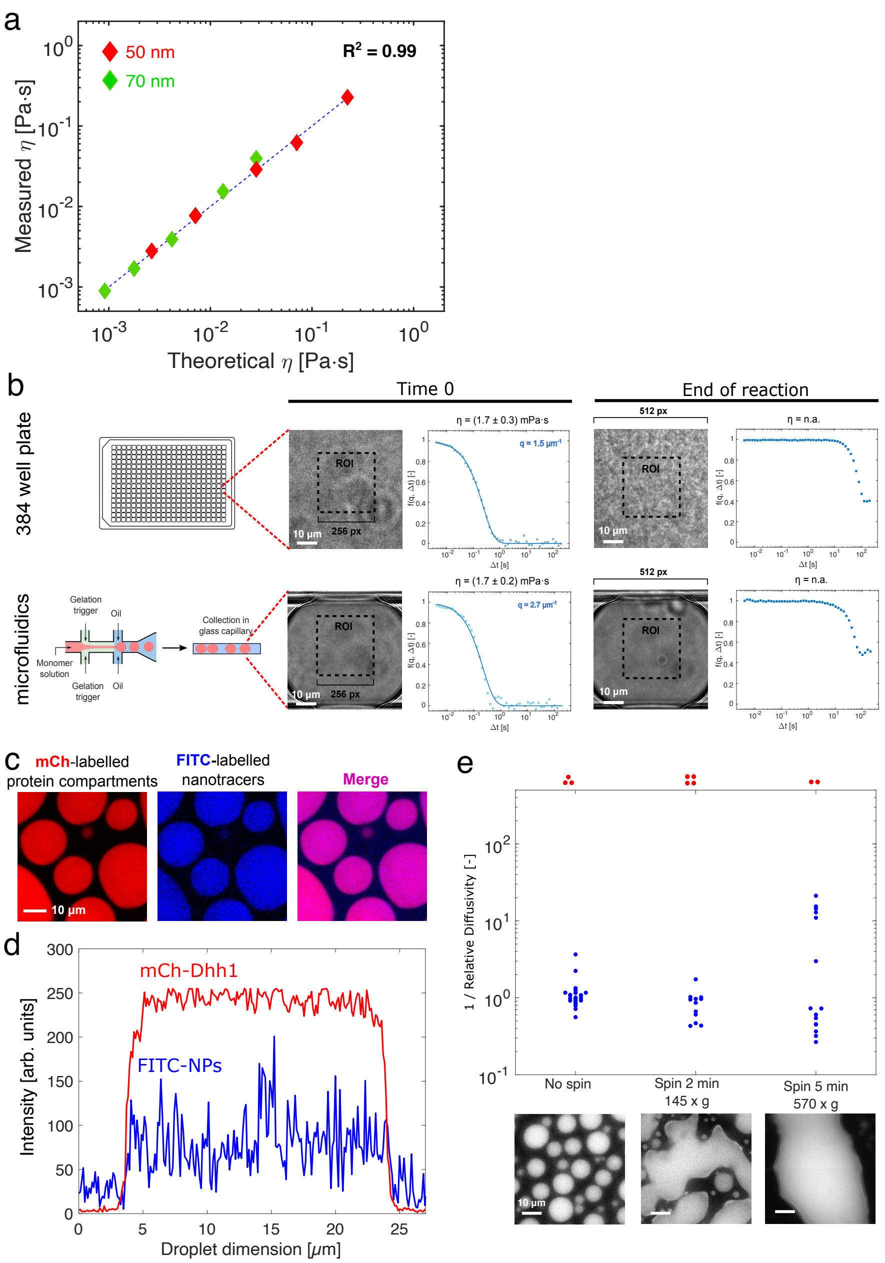

Suppl. Fig. 7: Validation and control experiments for the DDM technology. a DDM microrheology for the measurement of the viscosity of different water-glycerol mixtures with standard nanotracers (d = 50 nm, red; d = 70 nm, green). Comparison of experimentally measured viscosity with theoretical viscosities shows that DDM allows to measure viscosities precisely and over several orders of magnitude. **b** Absence of confinement effects on the diffusion of DDM nanotracers. The gelation of a polydimethylacrylamide (PDMA) hydrogel was monitored in bulk (384-well plate) and confined in microfluidic water-in-oil droplets) to mimic phase-separated condensates. Both at time 0 and at the end of the reaction, the ISFs were comparable, showing liquid-like and arrested states, respectively, independent of the size of the container in which it was carried out. **c** Internalization of 25 nm nanotracers into protein-rich droplets by confocal microscopy. The fluorescence of mCherry-tagged Dhh1 and FITC-coupled nanotracers overlap perfectly. **d** Intensity profiles of fluorescence intensity profiles of droplets. Red: mCherry-Dhh1, blue: 25 nm nanotracers. Tracers are regularly distributed throughout the droplets and are not locally up-concentrated. **e** Absence of confinement effects in condensates formed by Dhh1^{DQAD}. We generated compartments of increasing size by promoting the coalescence of Dhh1^{DQAD} condensates by centrifugation for either 2 minutes at 145 x g, or 5 minutes at 570 x g. Despite the evident differences in morphology and size the measured ISFs and relative diffusion coefficients are consistent. Source data of panels (a, d, e) are provided in the Source Data file.

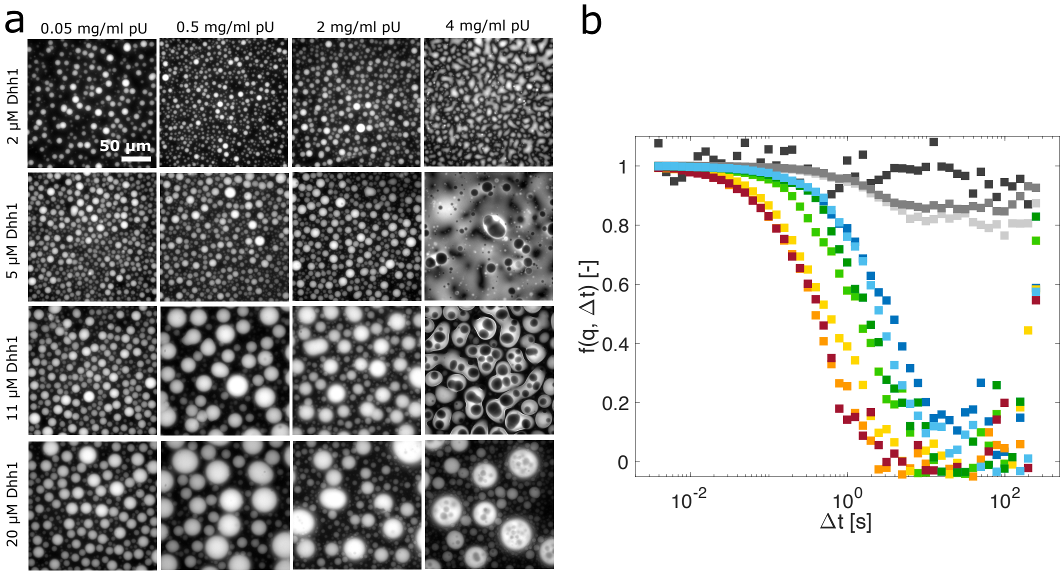

Suppl. Fig. 8: a Fluorescence microscopy images, taken at various ratios of Dhh1 to polyU. At high polyU concentrations, we observed vacuole formation, most likely corresponding to a re-entrant phase transition. **b** Intermediate Scattering Functions (ISFs) f(q, Δt) representing the decorrelation of ten droplets within the same sample. Liquid-like droplets are depicted in colors, dynamically arrested droplets in grey. Source data of panel (b) are provided in the Source Data file.

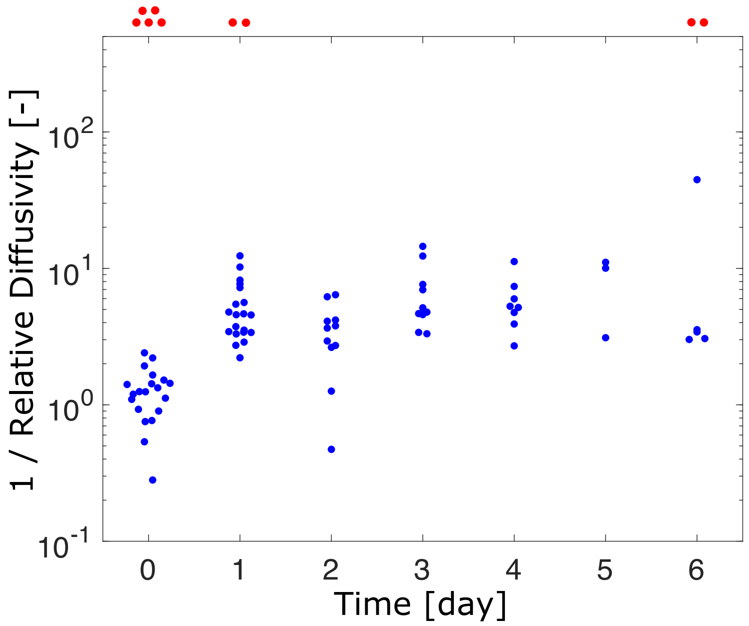

Suppl. Fig. 9: Droplets formed in presence of 10 mM ATP are still liquid-like on day 6, in contrast to droplets formed in presence of only 5 mM ATP. Source data of this figure are provided in the Source Data file.

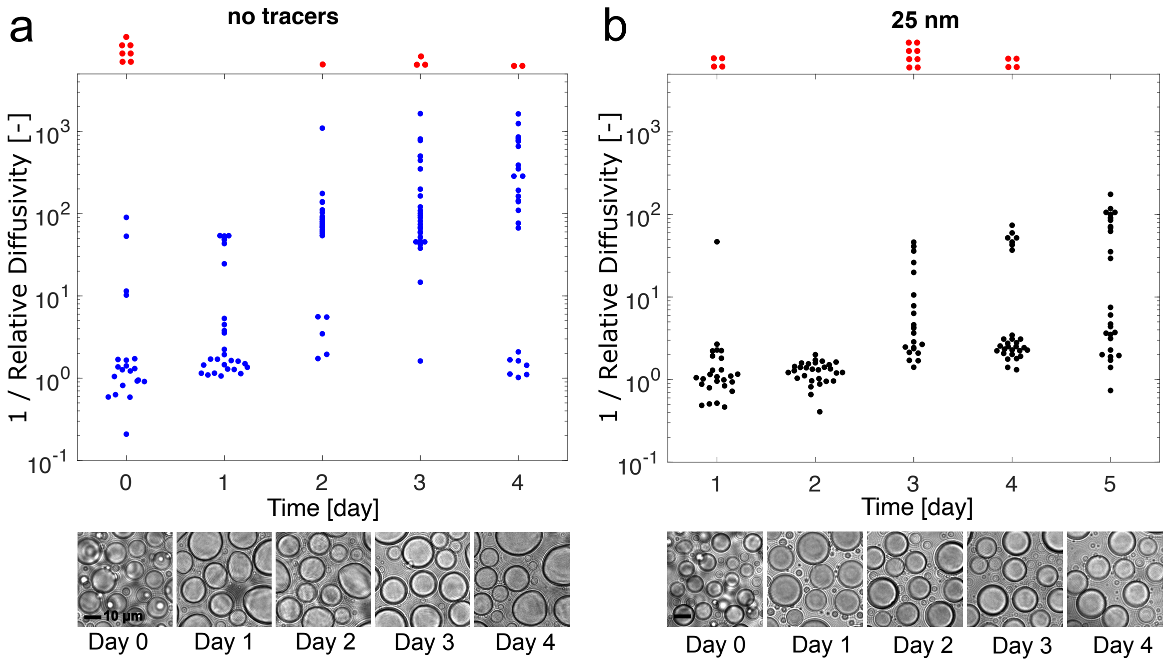

Suppl. Fig. 10: Absence of influence of the nanotracers on compartments aging. The aging of phase-separated Dhh1^{DQAD} compartments was investigated over 5 days in the absence of tracers (a) and in the presence of nanotracers with diameter of 25 nm (b). The results with and without tracers are consistent. For both panels, at least 10 droplets from 3 independent samples were measured. Source data of panels (a, b) are provided in the Source Data file.

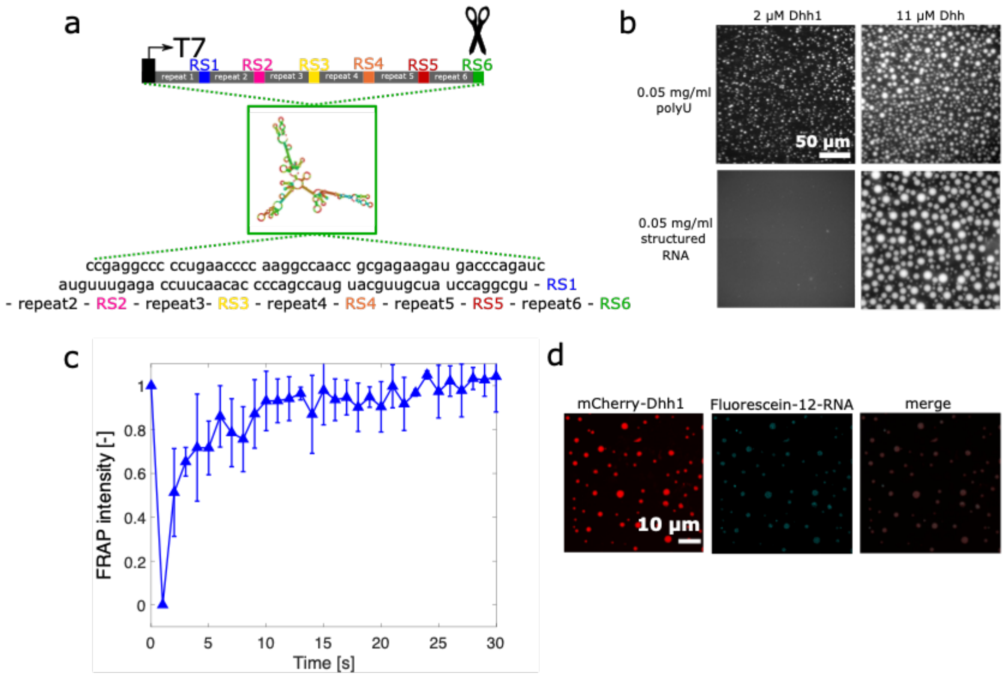

Suppl. Fig. 11: Effect of structured RNA on the phase separation propensity and material properties of Dhh1 condensates. **a** *In vitro* transcribed construct of repetitive 100 nucleotides of the actin mRNA sequence (sequence source) linked by six different restriction sites for six different restriction enzymes. To obtain the 600 nt RNA used in this study, the plasmid (pET-15b) was linearized by digestion with BamHI (RS6) and *in vitro* transcribed by T7 RNA polymerase. **b** Droplet formation in presence of unstructured polyU and structured RNA. In presence of 0.05 mg/ml polyU, droplets could be formed in presence of 2 µM Dhh1 and 5 mM ATP, whereas in presence of 0.05 mg/ml structured RNA 11 µM Dhh1 were required**. c** Mean fluorescence recovery after photobleaching (FRAP) signal of Dhh1 droplets formed in presence of 0.05 mg/ml polyU and 11 µM Dhh1 after 2 h of incubation shows that these droplets fully recover and thereby remain highly dynamic over the observed time scale. Error bars represent the standard deviation of three bleached droplets. **d** Droplet heterogeneity, shown by varying single droplet fluorescence intensity of mCherry-tagged Dhh1 and Fluorescein-12-labeled 600 nt structured RNA. Source data of panel (c) are provided in the Source Data file.
