# Supplementary material for: Dynamic arrest and aging of biomolecular condensates are modulated by low-complexity domains, RNA and biochemical activity
Source: Nat Commun. 2022 May 31;13:3030. doi: 10.1038/s41467-022-30521-2 (PMC9156751; doi:10.1038/s41467-022-30521-2)
Supplement: Supplementary file 2 — Reporting Summary [file 41467_2022_30521_MOESM2_ESM.pdf]

nature portfolio

Corresponding author(s): Paolo Arosio

Last updated by author(s): Apr 1, 2022

Reporting Summary

Nature Portfolio wishes to improve the reproducibility of the work that we publish. This form provides structure for consistency and transparency in reporting. For further information on Nature Portfolio policies, see our Editorial Policies and the Editorial Policy Checklist.

Statistics

For all statistical analyses, confirm that the following items are present in the figure legend, table legend, main text, or Methods section.

n/a	Confirmed	
☐	☒	The exact sample size (*n*) for each experimental group/condition, given as a discrete number and unit of measurement
☐	☒	A statement on whether measurements were taken from distinct samples or whether the same sample was measured repeatedly
☒	☐	The statistical test(s) used AND whether they are one- or two-sided<br>*Only common tests should be described solely by name; describe more complex techniques in the Methods section.*
☒	☐	A description of all covariates tested
☒	☐	A description of any assumptions or corrections, such as tests of normality and adjustment for multiple comparisons
☐	☒	A full description of the statistical parameters including central tendency (e.g. means) or other basic estimates (e.g. regression coefficient) AND variation (e.g. standard deviation) or associated estimates of uncertainty (e.g. confidence intervals)
☒	☐	For null hypothesis testing, the test statistic (e.g. *F*, *t*, *r*) with confidence intervals, effect sizes, degrees of freedom and *P* value noted<br>*Give P values as exact values whenever suitable.*
☒	☐	For Bayesian analysis, information on the choice of priors and Markov chain Monte Carlo settings
☒	☐	For hierarchical and complex designs, identification of the appropriate level for tests and full reporting of outcomes
☒	☐	Estimates of effect sizes (e.g. Cohen's *d*, Pearson's *r*), indicating how they were calculated

Our web collection on statistics for biologists contains articles on many of the points above.

Software and code

Policy information about availability of computer code

Data collection	Wide-field microscopy: Omicron Software (version 3.9.28), MicroManager (version 2.0 gamma)<br>Confocal microscopy: Leica Application Suite X (LAS X) (version 1.0)<br>Size exclusion chromatography: OpenLab ChemStation (version 0.0.1.98)<br>Dynamic Light Scattering: Zetasizer Nano software (version 7.13)<br>The data that support the findings of this study are available from the corresponding authors upon request. Source data are provided with this paper in the Source Data file.
Data analysis	Images were brightness-adjusted for illustrative purposes only, using Fiji / ImageJ (ImageJ2, version 2.3.0 / 1.53f)<br>Image analysis (extraction of droplet radii, total areas and volumes, intensities, FRAP analysis) was performed on raw images using an in-house written Matlab code (Matlab, version R2020a).<br>For DDM analysis we adapted a previously published Matlab code (GitHub, <dx.doi.org/10.5281/zenodo.30559>).<br>Adapted codes and all other used codes for data analysis are available from the corresponding authors upon request.

For manuscripts utilizing custom algorithms or software that are central to the research but not yet described in published literature, software must be made available to editors and reviewers. We strongly encourage code deposition in a community repository (e.g. GitHub). See the Nature Portfolio guidelines for submitting code & software for further information.

Data

Policy information about availability of data

All manuscripts must include a data availability statement. This statement should provide the following information, where applicable:

- Accession codes, unique identifiers, or web links for publicly available datasets
- A description of any restrictions on data availability
- For clinical datasets or third party data, please ensure that the statement adheres to our policy

All relevant data for the main text and supplementary information are provided in the source data file. Source data are provided with this paper. The raw data that support the findings of this study are available from the corresponding authors upon request.

Field-specific reporting

Please select the one below that is the best fit for your research. If you are not sure, read the appropriate sections before making your selection.

☒ Life sciences ☐ Behavioural & social sciences ☐ Ecological, evolutionary & environmental sciences

For a reference copy of the document with all sections, see nature.com/documents/nr-reporting-summary-flat.pdf

Life sciences study design

All studies must disclose on these points even when the disclosure is negative.

Sample size	For all experiments, 2-3 technical replicates were typically analyzed. No sample size calculation was performed. The sample size was chosen by feasibility with respect to protein production and computational speed.<br>For Dynamic Differential Microscopy analysis on a single droplet level, at least 10 droplets per replicate were analyzed.
Data exclusions	no data was excluded
Replication	We ensured reproducibility by repeating experiments using different recombinantly expressed and purified protein batches. All experiments were performed independently at least three times. All attempts at replication were successful. We carried out control experiments to compare our results and validate the DDM technique with results from literature / theory.
Randomization	Randomization was not performed as we are reporting on quantitative in vitro experiments and the samples were not allocated into experimental groups
Blinding	Blinding was not relevant for this study as all data analysis was performed in an automated, unbiased way, not influenced by the experimenter. Probed condensates were randomly chosen and could not be distinguished visually from each other, ensuring no introduction of bias.

Reporting for specific materials, systems and methods

We require information from authors about some types of materials, experimental systems and methods used in many studies. Here, indicate whether each material, system or method listed is relevant to your study. If you are not sure if a list item applies to your research, read the appropriate section before selecting a response.

Materials & experimental systems

n/a	Involved in the study	
☒	☐	Antibodies
☒	☐	Eukaryotic cell lines
☒	☐	Palaeontology and archaeology
☒	☐	Animals and other organisms
☒	☐	Human research participants
☒	☐	Clinical data
☒	☐	Dual use research of concern

Methods

n/a	Involved in the study	
☒	☐	ChIP-seq
☒	☐	Flow cytometry
☒	☐	MRI-based neuroimaging
